# Epidemiology of invasive pneumococcal disease in indigenous and non-indigenous adults in northwestern Ontario, Canada, 2006–2015

**DOI:** 10.1186/s12879-018-3531-9

**Published:** 2018-12-04

**Authors:** Daniel Dalcin, Lee Sieswerda, Sacha Dubois, Marina Ulanova

**Affiliations:** 1 Department of Medicine, Dalhousie University, 6299 South St, Halifax, NS B3H 4R2 Canada; 2 Northern Ontario School of Medicine, Thunder Bay, ON Canada; 3 Thunder Bay District Health Unit, Thunder Bay, ON Canada

**Keywords:** Immunocompromised, Indigenous, Invasive pneumococcal disease, Northwestern Ontario, Non-vaccine serotypes, Serotype distribution, *Streptococcus pneumoniae*, Vaccination

## Abstract

**Background:**

Despite the use of pneumococcal vaccines, indigenous populations are consistently disproportionately affected by invasive pneumococcal disease (IPD). With recent changes in Ontario’s provincial pneumococcal vaccination program, we sought to evaluate the epidemiology and burden of IPD in northwestern Ontario (NWO) Canada – a region that contains a substantial (19.2%) indigenous population.

**Methods:**

We retrospectively reviewed all adult cases of IPD that were reported to the Thunder Bay District Health Unit, in Thunder Bay, Ontario, Canada, over a 10-year period (2006–2015). Patients admitted to the Thunder Bay Regional Health Sciences Centre with IPD had their charts reviewed to abstract clinical data. Statistical analysis, including incidence rates of IPD, was performed.

**Results:**

Two hundred sixty-two cases of IPD occurred over the 10-year observation period and clinical data was available for 182 cases. Fifty-three of 182 (29.1%) patients were indigenous. 73 of 182 (40.1%) of patients were immunocompromised. Indigenous patients with IPD were more likely to be immunocompromised than non-indigenous patients (*p* < 0.001). Serotype data was available for 159 cases of IPD; PCV7, PCV13, and PPV23 covered 5.7%, 28.3%, and 79.2% of isolates, respectively, while 29 (20.8%) were non-vaccine serotypes. The annual incidence rate of IPD ranged from 8.9 to 25.9 per 100,000 among adults 18–64 years old; among adults 65 years of age and older the annual incidence of IPD ranged from 18.5 to 60.7 per 100,000.

**Conclusion:**

Among adults in NWO, Canada, there is a high incidence of IPD. Immunocompromised indigenous adults in NWO may benefit from pneumococcal vaccination coverage. Emerging non-vaccine serotypes of *Streptococcus pneumoniae* warrant the consideration of the provincial pneumococcal vaccination program.

## Background

*Streptococcus pneumoniae* causes substantial morbidity and mortality and remains an important cause of serious infection worldwide [1, 2]. From a global perspective the incidence of invasive pneumococcal disease (IPD) remains higher in indigenous populations compared to the general population despite the use of pneumococcal vaccines, but few studies have explored the burden of IPD in Canada’s indigenous population [3–9]. In Ontario, Canada, the provincial immunization program has seen recent changes, including the addition of pneumococcal conjugate vaccines (PCV7 in 2005, PCV10 in 2009, and PCV13 in 2010), yet the effectiveness of these vaccines in reducing clinical disease remains poorly explored.

In Canada, pneumococcal vaccination is currently recommend for all individuals (PCV13 at 2, 4, and 12 months; PPV23 at 65 years). In addition to routine pneumococcal immunization, additional PPV23 vaccination is currently recommended for individuals ≥24 months of age who have a medical condition or lifestyle risk factor that is considered high risk for IPD [10]. However, it seems that many Canadians do not receive the recommended pneumococcal immunizations. Public health surveillance of seven year old children that attended school in Ontario in the 2016–2017 year found that only 79.7% of children had received pneumococcal immunization [11]. Among adults, Statistics Canada estimates that in 2014 only 17.3% (13.7–20.8, 95% CI) of individuals 18–64 years of age with a chronic medical condition where PPV23 is indicated received at least one PPV23 immunization, compared to 36.5% (32.7–40.3, 95% CI) among adults 65 years of age and older [12].

The population of northwestern Ontario (NWO) is unique compared to most regions of North America in that it has a substantial indigenous population (19.2% of the population) dispersed over a vast geographic area [13]. In the context of recent changes in the provincial pneumococcal vaccination program, the objectives of our study were as follows: 1) to evaluate the incidence, trend of incidence, and serotype distribution of IPD in NWO; and 2) investigate differences in demographics, clinical characteristics, immune status, and serotype distribution of IPD among indigenous and non-indigenous adults in NWO.

## Methods

In Ontario, Canada, mandatory reporting legislation requires all cases of IPD to be reported to public health authorities; the Thunder Bay District Health Unit (TBDHU) receives mandatory reporting for NWO. We retrospectively reviewed all adult (individuals ≥18 years of age) cases of IPD reported to the TBDHU from 2006 to 2015 for age, gender, year of isolation, and serotype of *S. pneumoniae* causing IPD. Cases of IPD were defined as an isolation of *S. pneumoniae* from a sterile physiological site.

To further understand the demographic and clinical features of patients with IPD in NWO, we performed a chart review on a subset of patients identified from mandatory reporting to the TBDHU that were admitted to the Thunder Bay Regional Health Sciences Centre (TBRHSC). The TBRHSC is a 395 bed hospital and the largest hospital in NWO that serves as the primary referral hospital to the surrounding rural communities. To identify patients hospitalized with IPD, we performed an ICD-10 code search of “IPD” occurring between 2006 and 2015 from the TBRHSC health records that was independently corroborated by a search of the TBRHSC microbiology database for isolates of *S. pneumoniae* causing invasive disease.

Clinical and demographic data were retrospectively abstracted from patients’ charts. Patients were defined as immunocompetent or immunocompromised. Similar to other studies, we defined patients as immunocompromised if any of the following criteria was documented in patients’ chart: human immunodeficiency virus (HIV) infection, previous solid organ or bone marrow/stem cell transplantation, asplenia, sickle cell disease or other hematologic disorders, systemic lupus erythematosus, hematologic malignancy, hepatic cirrhosis, chronic renal failure (creatinine > 200 mg/dL or requiring chronic renal dialysis), primary immunodeficiency, or chronic receipt of immunosuppressive therapy. Immunosuppressive therapy was defined as chronic daily receipt of oral corticosteroids, current chemotherapy for cancer treatment, or other immunosuppressive medications used for chronic management of inflammatory/rheumatologic conditions [14].

Patients were defined as indigenous or non-indigenous. Indigenous status was defined as a patient with health insurance coverage provided by First Nations and Inuit Health and was available for 100% of patients with clinical data (*n* = 182). Mortality was defined as death within 30 days of isolation of *S. pneumoniae* from a physiologically sterile site. Serotyping was performed by the Quellung reaction using commercial pool, group, type and factor antisera at the time of microbiological diagnosis [15].

Statistical analysis was performed by using SAS version 9.3 (SAS Institute, Cary, North Carolina). Annual incidence rates were calculated based on population data provided from the Thunder Bay District Health Unit (TBDHU). Jointpoint regression, with annual percentage change (95% confidence interval), was used to evaluate changes in incidence rates over time. Statistical analysis was conducted to evaluate differences between indigenous and non-indigenous patients with IPD. Additionally, we compared case fatality rate between the indigenous and the non-indigenous stratifying by immune status, immunocompetent or immunocompromised. The Fisher exact test was used for binary variables (case-fatality, immune-status, indigenous status, gender, serotype distribution by vaccine coverage groups, co-morbidities) and the t-test was used for continuous variables (age). Statistical values were considered significant if *p* < 0.05.

## Results

Over the 10-year period (2006–2015), 262 adult cases of IPD were reported to public health (TBDHU) and had age, gender, year of isolation, and serotype data. 182 of the 262 cases (69.5%) involved patients hospitalized at the TBRHSC and had complete clinical and demographic data available. Of the 182 cases with clinical data, *S. pneumoniae* was isolated from the following sites: blood 164 (90.1%), cerebrospinal fluid 10 (5.8%), pleural fluid 5 (2.9%), synovial fluid 2 (1.2%), and peritoneal fluid 1 (0.6%).

The mean age of patients with IPD was 57.0 ± 18.6 years (range 21–97 years). During 2006–2015, the annual incidence rate of IPD ranged from 8.9 to 25.9 (16.9 average) per 100,000 among adults 18–64 years old; in adults 18 years of age and older the incidence ranged from 13.6 to 28.9 (37.7 average) per 100,000 (Table 1).

**Table 1:** Crude and age-standardized annual incidence rates (per 100,000 population) of invasive pneumococcal disease among adults in northwestern Ontario, Canada, by age group, 2006–2015

	18–64 years				> = 65 years				> = 18 years			
year	#	incidence	95% CI	# (%) male^a^	#	incidence	95% CI	# (%) male^a^	#	incidence	95% CI	# (%) male^a^
2006	12	11.7	6.64–20.6	6 (50.0)	10	43.8	23.6–81.4	5 (50.0)	22	17.5	11.6–26.7	11 (50.0)
2007	9	8.9	4.61–17.0	4 (44.4)	8	34.6	17.3–69.1	4 (50.0)	17	13.6	8.47–21.9	8 (47.1)
2008	17	16.9	10.5–27.1	9 (52.9)	7	29.7	14.1–62.2	4 (57.1)	24	19.3	12.9–28.7	13 (54.2)
2009	26	25.9	17.6–37.9	15 (57.7)	10	41.7	22.5–77.6	5 (50.0)	36	28.9	20.9–40.1	20 (61.1)
2010	15	14.9	9.0–24.7	7 (46.7)	13	53.5	31.1–92.1	6 (46.2)	28	22.4	15.5–32.5	13 (46.4)
2011	20	20.0	12.9–30.9	11 (55.0)	15	60.7	36.6–100.7	7 (46.7)	35	28.0	20.1–39.0	18 (51.4)
2012	17	17.0	10.6–27.4	10 (58.8)	7	27.4	13.1–57.5	3 (42.9)	24	19.1	12.8–28.6	13 (54.2)
2013	18	18.1	11.4–28.8	9 (50.0)	11	41.7	23.1–75.4	5 (45.5)	29	23.1	16.1–33.2	14 (48.2)
2014	18	18.3	11.5–29.0	10 (55.6)	5	18.5	7.72–44.6	5 (100.0)	23	18.3	12.2–27.6	15 (65.2)
2015	17	17.4	10.8–27.9	11 (64.7)	7	25.4	12.1–53.2	3 (42.9)	24	19.1	12.8–28.6	14 (58.3)

Age-adjusted crude incidence rates were calculated based on annual population data provided by the Thunder Bay District Health Unit

^a^ Percentage female implied as difference from 100%

Fifty-three of 182 (29.1%) patients were indigenous; 73 of 182 (40.1%) were immunocompromised. Thirty-five of 53 (66.0%) indigenous and 38 of 129 (29.5%) non-indigenous patients were immunocompromised (*p* < 0.001, Fisher exact test). The average age of indigenous and non-indigenous adults with IPD was 43.4 and 62.7 years, respectively, and the difference was statistically significant (p < 0.001).

Overall, a wide range of immunocompromising conditions were observed in both indigenous and non-indigenous patients with IPD. The most common cause of immunodeficiency in indigenous patients with IPD was receipt of chronic immunosuppressive therapy (8 patients, 22.9%) compared to a lower rate observed in non-indigenous patients (2 patients, 5.3%), and the difference was statistically significant (Fisher exact test, *p* = 0.041). Hematological malignancy was found to be a more common immunocompromising condition among the non-indigenous cohort (10 patients, 26.3%) compared to the indigenous cohort (1 patient, 2.9%) and the difference was statistically significant (Fisher exact test, *p* = 0.007). Among the immunocompromised patients with IPD, HIV infection was a more common immunocompromising condition observed in non-indigenous patients compared to indigenous patients (1 case, 2.9%; 6 cases, 15.8%, respectively), but the difference was not statistically significant.

There were 4 deaths among the 53 indigenous patients compared to 11 deaths among the 120 non-indigenous patients (case-fatality rate of 7.5% indigenous versus 8.5% non-indigenous; not statistically significant difference). A case-fatality rate in non-indigenous immunocompromised patients (5 deaths/38 patients, 13.2%) exceeded one in indigenous immunocompromised patients (1 death/35 patients, 2.86%), but the difference was not statistically significant.

**Fig. 1:**
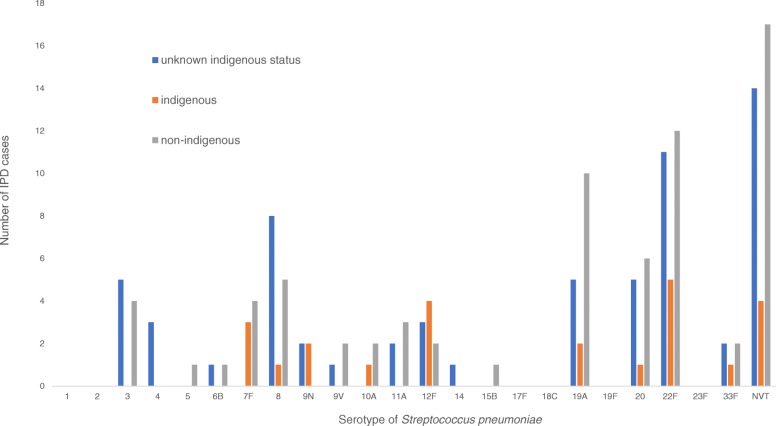
Serotype distribution of 159 isolates of Streptococcus pneumoniae causing invasive pneumococcal disease among adults in northwestern Ontario, Canada, 2006-2015, by indigenous status. Non-vaccine serotypes accounted for 20.8% of *S. pneumoniae* serotypes causing invasive pneumococcal disease during the 10-year observation period

Serotype data was available for 159/262 (60.7%) of IPD cases (Fig. 1). The most common serotypes causing IPD were 22F (17.6%), 19A (12.6%), 20 (8.8%), and 8 (7.5%), which accounted for 46.5% of all typed isolates. PCV7, PCV13, and PPV23 covered 5.7, 28.3, and 79.2% of isolates, respectively, while 29 (20.8%) were non-vaccine serotypes (NVT).

The serotypes of *S. pneumoniae* isolates causing IPD in indigenous patients (serotype data were available for 22 of 53 cases) were covered by the following pneumococcal vaccines: PCV7 (0, 0%), PCV13 (4, 18.2%), PPV23 (14, 63.6%), NVT (4, 18.2%). There was no statistically significant difference among *S. pneumoniae* serotypes (according to vaccine coverage group: PCV7, PCV13, PPV23, NVT) causing IPD between indigenous and non-indigenous patients.

The serotypes of *S. pneumoniae* causing IPD in immunocompromised patients (serotype data were available for 48 of 73 cases) were covered by the following pneumococcal vaccines: PCV7 (1, 2.1%), PCV13 (13, 27.1%), PPV23 (22, 45.8%), NVT (12, 25.0%). There was no statistical difference among *S. pneumoniae* serotypes (according to vaccine coverage group: PCV7, PCV13, PPV23, NVT) causing IPD between immunocompetent and immunocompromised patients.

## Discussion

Our study found a high incidence of IPD among adults in NWO. During the same period of observation, the national IPD incidence rates ranged between 8.85 and 9.88 per 100,000 population of all ages, including children [16], while the annual IPD incidence rates from NWO were 13.6–28.9 per 100,000 for adults ≥18 years of age (Table 1). In particular, the incidence rates of IPD in ≥65 year-old adults in NWO were higher than recently reported data from the same age group in the whole province of Ontario (2007–2014), i.e. 37.7/100,000 versus 25.4/100,000 [17].

Our findings demonstrate that indigenous adults are disproportionately represented among individuals hospitalized for IPD at the TBRHSC. While the indigenous people of NWO represent 19.2% of the population, 29.1% of patients hospitalized for IPD at the TBRHSC were identified as indigenous. We also found that indigenous patients hospitalized at the TBRHSC with IPD were statistically more likely to be immunocompromised than non-indigenous patients. Taken together, these findings suggest that the higher rate of comorbid conditions causing immunodeficiency among the indigenous population may contribute to the burden of IPD in NWO.

Although 66% of indigenous patients with IPD were immunocompromised (compared to 29.5% of non-indigenous patients with IPD, *p* < 0.001) there was no statistical difference in case-fatality rate between indigenous and non-indigenous patients (7.5 and 8.5%, respectively). This difference in case-fatality is a striking difference from the findings of an Ontario population-based monitoring study where immunocompromised individuals with IPD were documented to have case-fatality rates of 16.2% in individuals 15–64 years of age and 29.5% in individuals 65 years of age and older [14]. The average age of indigenous patients was 19.3 years younger than non-indigenous patients which may have contributed to the lower case-fatality rate (Table 2).

**Table 2:** Age, gender, immune-status, and case-fatality rate by indigenous status among 182 adults hospitalized for IPD in northwestern Ontario, Canada, 2006–2015

	No. of patients (%)	Mean age, years (standard deviation)	Gender		Immune status		
					Immuno-compromised (%)	Immuno-competent (%)	Total
			Male (%)	Female (%)			
Indigenous	53 (29.1)	43.4 (17.9)	31 (58.4)	22 (41.5)	35 (66.0)	18 (34.0)	53
Death count					1 (25.0)	3 (75.0)	4
Case-fatality rate (%)					2.9	16.7	7.5
Non-indigenous	129 (70.9)	62.7 (14.3)	73 (56.6)	56 (43.4)	38 (29.5)	91 (70.5)	129
Death count					5 (45.5)	6 (54.5)	11
Case-fatality rate (%)					13.2	6.6	8.5
*p* values*		< 0.001	0.58	0.35	0.21	0.17	1.00

*p values for difference between indigenous and non-indigenous values. Statistical analysis: Fisher Exact test (gender, death counts among immunocompromised, immunocompetent, and total); t-test (mean age). α = 0.05

Our study did not identify statistically significant differences in *S. pneumoniae* serotype distribution among non-indigenous, indigenous, or immunocompromised patients with IPD. However, our findings of a relatively high prevalence of PPV23 serotypes suggests inadequate vaccination of high-risk indigenous adults (Fig. 1). A larger study is required to evaluate this possibility, which has been observed in other indigenous populations [18].

The occurrence of serotypes 22F (17.6%) and 8 (7.5%), which have been recognized as emerging serotypes in the post-PCV13 era, is worrisome; along with a high proportion of NVT (20.8%), this implies serotype replacement [19, 20]. The fact that the serotype 19A (included into PCV13) accounts for 12.6% of IPD cases in our population may indicate insufficient herd effect of pediatric immunization with PCV13; the latter was introduced in Ontario in 2010 [16].

Our study has limitations. Similar to other retrospective studies, we were unable to obtain complete and accurate vaccination data. Therefore, we were unable to comment on individual cases of vaccine failure or differences in vaccine coverage between indigenous and non-indigenous individuals. Outside of the cases of IPD occurring at the TBRHSC, our data relies on mandatory reporting to public health. Although most clinical microbiological laboratories in Ontario have well-established mandatory reporting algorithms, cases of IPD not reported to public health would not be captured in our study and underestimate the incidence of IPD in this region. The clinical data obtained in our study only evaluated patients hospitalized with IPD at the TBRHSC and did not include patients who were not hospitalized at the TBRHSC or patients admitted to surrounding community hospitals. Therefore, our clinical data may not be representative of the population in terms of disease severity, indigenous status, or immunocompromised status.

## Conclusions

The findings of this study provide valuable data on the effectiveness of the Ontario pneumococcal vaccination program in a region with a substantial indigenous population over a critical time period where changes were made in the provincial pneumococcal immunization program. Our study documents a high incidence of IPD among adults in NWO, Canada. Additionally, we found that among patients with IPD hospitalized at the TBRHSC, the indigenous population was disproportionately affected. In particular, our findings identify immunocompromised indigenous adults in NWO, Canada as a vulnerable cohort of the population that may benefit from pneumococcal vaccination coverage. Emerging NVTs warrant consideration from the provincial pneumococcal vaccination program. These findings serve as an impetus for public health action.

## Abbreviations

CI: Confidence interval
HIV: Human immunodeficiency virus
IPD: Invasive pneumococcal disease
NVT: Non-vaccine serotype
NWO: Northwestern Ontario
PCV7: Pneumococcal conjugate vaccine 7
PCV13: Pneumococcal conjugate vaccine 13
PPV23: Pneumococcal polysaccharide vaccine 23
TBDHU: Thunder Bay District Health Unit
TBRHSC: Thunder Bay Regional Health Sciences Centre

## References

[1] Drikoningen JJ, Rohde GG (2014). Pneumococcal infection in adults: burden of disease. Clin Microbiol Infect.

[2] GBD 2016 Lower Respiratory Infections Collaborators. Estimates of the global, regional, and national morbidity, mortality, and aetiologies of lower respiratory infections in 195 countries, 1990–2016: a systematic analysis for the Global Burden of Disease Study 2016, Lancet Infect Dis. 2018;S1473-3099(18)30310-4. 10.1016/S1473-3099(18)30310-4 PMC6202443 30243584

[3] Degani N, Navarro C, Deeks SL, Lovgren M (2008). Invasive bacterial diseases in northern Canada. Emerg Infect Dis.

[4] Bruce MG, Deeks SL, Zulz T, Bruden D, Navarro C, Lovgren M (2008). International circumpolar surveillance system for invasive pneumococcal disease, 1999-2005. Emerg Infect Dis.

[5] Lehmann D, Willis J, Moore HC, Giele C, Murphy G, Keil AD (2010). The changing epidemiology of invasive pneumococcal disease in aboriginal and non-aboriginal Western Australians from 1997 through 2007 and emergence of non-vaccine serotypes. Clin Infect Dis.

[6] Segal N, Greenberg D, Dagan R, Ben-Shimol S (2016). Disparities in PCV impact between different ethnic populations cohabiting in the same region: a systematic review of the literature. Vaccine.

[7] Ya L, Martin I, Tsang R, Squires SG, Demczuk W, Desai S (2016). Invasive bacterial diseases in northern Canada, 2006-2013. Can Comm Dis Rep.

[8] Singleton RJ, Hennessey TW, Bulkow LR, Hammitt LL, Zulz T, Hurlburt DA (2007). Invasive pneumococcal disease caused by non-vaccine serotypes among Alaska native children with high levels of 7-valent pneumococcal conjugate vaccine coverage. JAMA.

[9] Weatherholtz R, Millar EV, Moulton LH (2010). Invasive pneumococcal disease a decade after a pneumococcal conjugate vaccine use in an American Indian population at high risk for disease. Clin Infect Dis.

[10] Canadian Immunization Guide: Part 4 – Active Vaccines. <https://www.canada.ca/en/public-health/services/publications/healthy-living/canadian-immunization-guide-part-4-active-vaccines/page-16-pneumococcal-vaccine.html#risk-factors>. Accessed September 29, 2018.

[11] Public Health Ontario. Immunization coverage report for school pupils in Ontario; 2016–17 school year. < https://www.publichealthontario.ca/en/eRepository/immunization-coverage-report-2016-17.pdf>. Accessed September 29, 2018.

[12] Vaccine uptake in Canadian adults: results from the 2014 adult National Immunization Coverage Survey. < https://www.canada.ca/en/public-health/services/publications/healthy-living/vaccine-uptake-canadian-adults-results-2014-adult-national-immunization-coverage-survey.html>. Accessed September 29, 2018.

[13] Northwest LHIN Environmental Scan, 2013. <http://www.tbrhsc.net/wp-content/uploads/2018/05/2013-Spring-North-West-LHIN-Common-Environmental-Scan.pdf>. Accessed October 28, 2018.

[14] Shigayeva A, Rudnick W, Green K, Chen DK, Demczuk W, Gold WL (2016). Invasive pneumococcal disease among immunocompromised persons: implications for vaccination programs. Clin Infect Dis.

[15] Slotved HC, Kaltoft M, Skovsted IC, Kern MB, Espersen F (2004). Simple, rapid latex agglutination test for serotyping of pneumococci (Pneumotest-latex). J Clin Microbiol.

[16] Invasive pneumococcal disease, Notifiable Diseases Online (2017). Public Health Agency of Canada.

[17] Desai S, Policarpio ME, Wong K, Gubbay J, Fediurek J, Deeks S (2016). The epidemiology of invasive pneumococcal disease in older adults from 2007 to 2014 in Ontario, Canada: a population based study. CMAJ Open.

[18] Moberley S, Krause V, Cook H, Mulholland K, Carapetis J, Torzillo P (2010). Failure to vaccinate or failure of vaccine? Effectiveness of the 23-valent pneumococcal polysaccharide vaccine program in indigenous adults in the Northern Territory of Australia. Vaccine.

[19] Duvvuri VR, Deng X, Teatero S, Memari N, Athey T, Fittipaldi N (2016). Population structure and drug resistance patterns of emerging non-PCV-13 *Streptococcus pneumoniae* serotypes 22F, 15A, and 8 isolated from adults in Ontario, Canada. Infect Genet Evol.

[20] Golden AR, Adam HJ, Zhanel GG, Alliance CAR (2016). Invasive *Streptococcus pneumoniae* in Canada, 2011–2014: Characterization of new candidate 15-valent pneumococcal conjugate vaccine serotypes 22F and 33F. Vaccine.

